# Rebound of *Cotton leaf curl Multan virus* and its exclusive detection in cotton leaf curl disease outbreak, Punjab (India), 2015

**DOI:** 10.1038/s41598-017-17680-9

**Published:** 2017-12-12

**Authors:** Sibnarayan Datta, Raghvendra Budhauliya, Bidisha Das, Reji Gopalakrishnan, Sonika Sharma, Soumya Chatterjee, Vanlalhmuaka, P. Srinivas Raju, Vijay Veer

**Affiliations:** 1 Molecular Virology Laboratory, Biotechnology Division, Defence Research Laboratory (DRL-DRDO), Tezpur, 784 001 India; 2 Present Address: Vector Management Division, Defence Research & Development Establishment (DRDE-DRDO), Gwalior, 474 002 India

## Abstract

Cotton leaf curl disease (CLCuD) outbreaks caused by CLCuD associated begomoviruses (CABs) significantly constrain cotton production in India and Pakistan. In comparison to the CABs circulating in Pakistan, molecular epidemiology, evolution and recombination patterns of CABs circulating in India are less studied. In this work, we characterized CAB complex sequences obtained from the most recent outbreak (Punjab, India, 2015), and rigorously analyzed them with reference to GenBank sequences, submitted from India, Pakistan and other neighbouring countries, using contemporary bioinformatics approaches. In this manuscript, we illustrate the detection of a recombinant, phylogenetically distinct clade of *Cotton leaf curl Multan virus* (CLCuMuV), suggesting rebound of CLCuMuV in this region. Interestingly, we could not detect *Cotton leaf curl Kokhran virus*-Burewala strain (CLCuKoV-Bu), which was prevalent in this region, until now. Our study thus indicates substitution of the ‘*virulent resistance breaking’* CLCuKoV-Bu by the re-emerging CLCuMuV recombinants. Our findings corroborate with that of a very recent study from Pakistan and we here discuss epidemiological links between the CAB complexes reported in these two studies. Taken together, these observations signify a shifting epidemiology of CABs, and seem to correlate with the recent prediction of the ‘*third epidemic*’ of CLCuD in the Indian subcontinent.

## Introduction

Worldwide, cotton is considered as the most important non-food agricultural commodity and is one of the major economic crops of the Indian and the African subcontinents. Production of cotton in these two subcontinents is severely constrained by cotton leaf curl disease (CLCuD), which is considered as the most complex and economically important disease of cotton^1–3^. The etiological viral agents associated with this disease are collectively known as CLCuD associated begomoviruses (CABs), which belong to the genus *Begomovirus* (family *Geminiviridae*), and are predominantly transmitted by ubiquitous whitefly (*Bemisia tabaci*)^3–5^. The genome of the CABs predominantly consists of a monopartite circular ssDNA (denoted as DNA-A)^3,5^, frequently associated with non-viral, single stranded circular satellite DNA molecules (alphasatellite and/or betasatellite), together presenting as an infection complex^3,6,7^. Though the role of alphasatellite is still enigmatic, betasatellite is instrumental in determining disease severity, symptoms and adaptability to the host^8,9^. Evolution of the CABs take place via frequent interspecific and/or intraspecific recombinations, which play an important role in emergence/re-emergence of recombinants having increased virulence, transmissibility and ability to overcome host resistance, eventually leading to epidemics^1,3,10,11^.

From the perspective of molecular epidemiology, distinct species of CLCuD complexes are endemic to the African and the Indian subcontinents^3,8^. Till date, African complex consisted of a single species of monopartite begomovirus, an alphasatellite and a betasatellite, the *Cotton leaf curl Gezira virus* (CLCuGeV), *Cotton leaf curl Gezira alphasatellite* (CLCuGeA) and the *Cotton leaf curl Gezira betasatellite* (CLCuGeB), respectively^3,12^. However, very recently, *Cotton yellow mosaic virus* (CYMV), a bipartite begomovirus was reported to infect cultivated cotton in Benin, West Africa^13^. In sharp contrast, high genetic diversity of CABs has been documented in cotton growing areas across the Indian subcontinent^1,3,6,9,10^. The high genetic diversity of CABs corroborate with the diversity of other geminiviruses in this region, since the Indian subcontinent is considered as the centre for geminivirus origin, evolution and diversity^6,14,15^. In this region, at least four closely related CABs, namely the *Cotton leaf curl Alabad virus* (CLCuAlV), *Cotton leaf curl Bangalore virus* (CLCuBaV), *Cotton leaf curl Kokhran virus* (CLCuKoV) and the *Cotton leaf curl Multan virus* (CLCuMuV) have been identified as ‘core’ viruses, while CLCuGeV has been reported to be a recent introduction into this region^3,8^. Additionally, a number of other ‘non-core’ geminiviruses have also been reported to infect cotton in the Indian subcontinent, including *Papaya leaf curl virus* (PaLCuV), *Chickpea chlorotic dwarf virus* (CpCDV), *Okra enation leaf curl virus* (OEnLCV), *Tomato leaf curl Bangalore virus* (ToLCuBaV), and *Tomato leaf curl New Delhi virus* (ToLCNDV)^3,6,8^. Despite the diversity of CABs, CLCuMuV and CLCuKoV or their strains such as *Cotton leaf curl Multan virus* - Rajasthan (CLCuMuV-Ra) and *Cotton leaf curl Kokhran virus* - Burewala (CLCuKoV-Bu) are predominantly associated with episodes of devastating CLCuD epidemics in the Indian subcontinent^3,8,16^. CLCuMuV-Ra and CLCuKoV-Bu have been shown to have evolved due to interspecies recombination between CLCuMuV and CLCuKoV^3^. Interestingly, among the CABs prevalent in the Indian subcontinent, CLCuBaV and CLCuMuV-Ra are believed to have evolved in India, while most of the other species and recombinants are thought to have evolved in Pakistan, and have consequently spread into the adjoining regions of northwestern India^3,6,9,10^. Likewise, diverse alphasatellite molecules including *Cotton leaf curl Multan alphasatellite* (CLCuMuA), *Cotton leaf curl Burewala alphasatellite* (CLCuBuA), *Cotton leaf curl Shadadpur alphasatellite* (CLCuShA), *Gossypium darwinii symptomless alphasatellite* (GDarSLA), *Gossypium davidsonii symptomless alphasatellite* (GDavSLA), *Gossypium mustilinum symptomless alphasatellite* (GMusSLA) have been identified in cotton^1,3,10^. Nevertheless, in sharp contrast to the begomovirus and alphasatellite diversity, *Cotton leaf curl Multan betasatellite* (CLCuMuB) has been identified as the predominant betasatellite circulating in this region^6,9,10,17^.

The first outbreak of CLCuD in the Indian subcontinent, the ‘*Multan epidemic*’ occurred in Multan, Khanewal and Vehari districts of Punjab province of Pakistan during the 1990s which was later found to be associated with a begomovirus-betasatellite complex comprised of CLCuMuV, CLCuKoV, CLCuAlV, PaLCuV along with a single betasatellite, the CLCuMuB^1,3,6^. This outbreak was consequently controlled by large scale adoption of CLCuD resistant cotton varieties^3,6,8^. Although, the adoption of resistance varieties helped in control of the disease, it also paved way for evolution and emergence of the virulent, resistance breaking CLCuKoV-Bu, leading to the ‘*Burewala epidemic*’ during 2000s in Burewala, Vehari district of Punjab province of Pakistan, hallmarking the second epidemic of CLCuD in the subcontinent^1,3,9,10,18^. In India, initial outbreak of CLCuD was reported in 1994 from Sri Ganganagar (Rajasthan state of India), adjoining Pakistan, which spread rapidly into Punjab (India) and to rest of the north Indian cotton growing zone within 4–5 years^3,6,19–23^. A drastic shift in the CLCuD molecular epidemiology in India was indicated during 2004–2005, when cotton crops that were earlier resistant to CLCuD, suddenly became susceptible^20–24^. Sequence analyses later clarified that CLCuMuV-Ra was the predominant CAB in the cotton fields of northwestern India until 2004–2005, which dramatically changed in the subsequent years with the introduction, establishment and predominance of the resistance breaking CLCuKoV-Bu in India^3,21^. CLCuKoV-Bu was also associated with the 2009–10 severe outbreak of CLCuD causing almost complete destruction of cotton crops in Punjab and Rajasthan states of India^20–24^. Since then CLCuKoV-Bu has remained as the single dominant CAB, associated with CLCuD in India and Pakistan^1,3,8^.

During the cotton cropping season of 2015–16, massive infestation of whiteflies was observed in the cotton fields of southern Punjab (India), followed by manifestation of CLCuD in an epidemic mode^2,25,26^. According to the reports and statistics, more than 2/3^rd^ of the cotton crop cultivated in nearly 11.5 Lakh acres was completely destroyed, sharply declining the productivity (from 21 Lakh Bales during previous years to merely 9 Lakh Bales predicted for the year 2015–16), causing an estimated loss worth 630–670 million US dollars^27–29^. Consequently, cotton plantation area also reduced by 40% during the subsequent crop year (i.e., 2016–17)^27^. Apart from shattering the economy from farmer house-hold to the national level, this devastating outbreak also claimed lives of at least 15 cotton farmers, who committed suicide due to heavy losses, triggering serious socio-political turmoil in the state^28^.

Very interestingly, in field trials conducted by the Indian Council of Agricultural Research (ICAR) at its different field stations in the northern and northwestern India (New Delhi, Punjab, Rajasthan and Haryana), a number of cotton cultivars, which were found to be highly resistant to CLCuD during crop season 2011–2012, showed susceptible to highly susceptible reaction to CLCuD under unprotected natural field/green house conditions during the subsequent crop seasons (2013–14)^26,30^. In these experiments, almost all the varieties of Bt hybrid cotton, which were earlier claimed to be resistant or tolerant to CLCuD were found to be susceptible to highly susceptible. Correlating these observations with the recent CLCuD outbreak in Punjab, we hypothesized that the etiological virus associated with the 2015 Punjab outbreak was divergent from the CLCuKoV-Bu, which is known to be the predominant CAB in this region. To examine our hypothesis, we collected plant samples directly from outbreak stricken cotton fields of Punjab, determined the complete genome of the associated begomoviruses, alphasatellite and betasatellites, performed rigorous phylogenetic and recombination analyses. In this manuscript, we report very important and interesting findings of this study, which endows with an exciting insight into the ongoing changing molecular epidemiology of the CABs in the Indian subcontinent. We subsequently discuss the significance of our findings with reference to the recent literature.

## Materials and Methods

### Sample collection and DNA extraction

**Figure 1:**
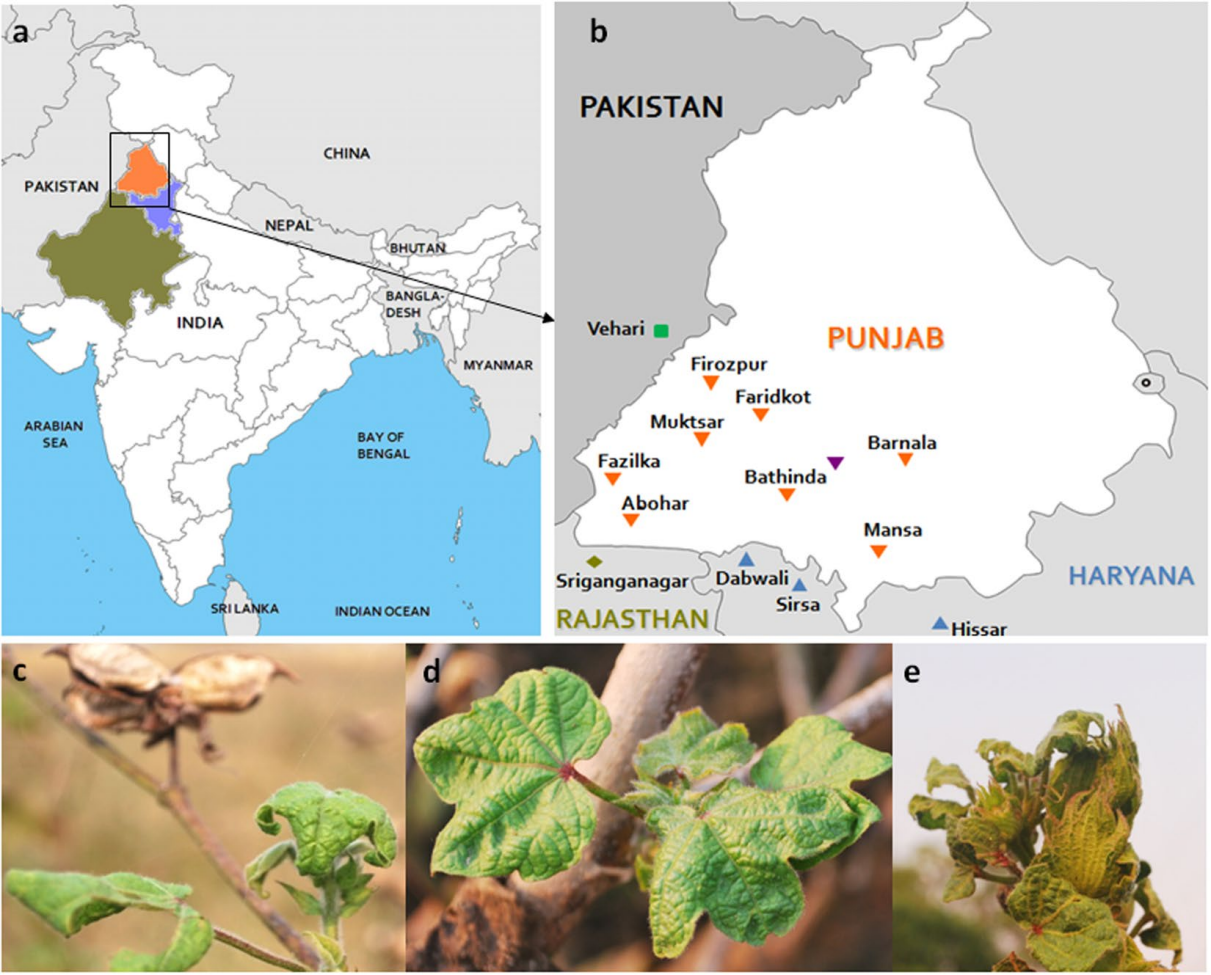
Figure showing the three cotton growing states of northwestern India, namely Punjab (shown in saffron), Rajasthan (Olive), Haryana (blue) and their adjacency to Pakistan (**a**). Different cotton growing areas of southern Punjab that were severely affected by the present outbreak are shown in panel b. Other cotton growing areas of Rajasthan and Haryana, neighbouring Punjab are also shown in panel (b). Some of the symptoms observed on cotton plants are shown in panels c to e. Base maps were acquired from the d-maps website (http://d-maps.com/carte.php?num_car=4182&lang=en and http://d-maps.com/carte.php?num_car=9012&lang=en); annotated, and the final representation was created with the help of the Microsoft Office PowerPoint 2007 and Microsoft Windows Paint (version 1607) softwares.

Due to widespread socio-political turmoil in the state, we were able to access the affected farmer’s fields for sample collection only during the last week of October, 2015. Unfortunately, by this time, most of the farmers have already ploughed back their crops to make their fields ready for the next cultivation. Nevertheless, discussion with local farmers helped us to locate certain pocket in the Barnala district of Punjab (30°17′53″N 75°22′08″E), where severely affected cotton plants were still in the fields. Location of Punjab, and areas worst affected by this outbreak are shown in Fig. 1. We surveyed 10 such cotton fields and collected 50 leaf samples with severe symptoms of CLCuD (downward curling of leaves, vein thickening & yellowing, stunted plant growth etc. (Fig. 1 and Supplementary Fig. S1). Samples were subsequently preserved and transported to the laboratory for further processing. On reaching the laboratory, 20 leaf samples (2 samples from each of the 10 sampled fields) were processed and total DNA was extracted using a commercially available plant DNA extraction kit (Sigma-Aldrich, St. Louis, USA). Quality and quantity of the extracts were checked on agarose gels and spectrophotometer (Picodrop, Cambridge, UK), respectively. In addition to the cotton leaf samples, two samples from unrelated plants (without any begomoviral infection, previously verified by RCA-PCR) and one sample with *Tomato leaf curl Bangladesh virus* (ToLCBV) and *Tomato leaf curl Bangladesh betasatellite* (ToLCBB) infection^31^, collected from a distant geographical location (Tezpur, Assam, India, 26.63°N, 92.8°E) were also processed concurrently throughout the experiments. These samples served as controls during the entire experimental procedures.

### Rolling Circle Amplification (RCA) and Polymerase Chain Reaction (PCR)

Total DNA extracts were diluted and subsequently subjected to Φ29 DNA polymerase (Fermentas, Villinius, Lithuania) mediated RCA to enrich the begomoviral and satellite DNA components. RCA products were partial digested by incubation with 1 U of restriction endonuclease *Hind* III (Sigma-Aldrich) for 25 mins at 37 °C following Khan *et al*.^32^, column purified (Sigma-Aldrich), quantified (Picodrop) and diluted to approximately 50 ng/µL. Enriched RCA products were subsequently used in individual PCR assays for partial or complete amplification of each of the four genetic components, namely the DNA-A, DNA-B, alphasatellite and betasatellite, using previously reported begomovirus complex universal primer pairs^1,33,34^ for DNA-A (DengA/ DengB, CRv301/CRc1152, PAR1c496/PAL1v1978 and PAR1v722/PAL1c1960), DNA-B (BF518/BR1641), alphasatellite (DNA101/DNA102) and betasatellite (Beta01/Beta02). DNA samples amplifiable with universal DNA-A primer pairs were further amplified with additional overlapping primer pairs to sequence the entire genome of the begomoviruses. Details of the PCR primers designed in the present study can be found online as Supplementary Table S1, Fig. S2. Guidelines and precautions to avoid cross contamination during RCA and PCR set-ups and amplicon analyses were strictly followed throughout the experiments^35^.

### Cloning and Sequencing

Amplicons generated in individual PCR reactions were column purified (Sigma-Aldrich), ligated into T/A cloning vector (Fermentas) and were used to transform XL1-Blue competent bacterial cells. Five-six transformed clones were randomly selected from each transformation experiment, cultured and plasmid DNA was purified (Sigma-Aldrich). Two clones each of the two amplicons denoting subgenomic betasatellites were also prepared. Each plasmid DNA was then diluted and PCR amplified using vector specific primers. PCR amplicons were finally sent to commercial facilities for sequencing (BioServe Biotechnologies, Hyderabad and SciGenom Labs, Kochi, India).

### Sequence analysis

Sequence electropherograms were manually examined, edited and vector sequence trimmed using Bioedit program version 7.2.5^36^. Sequences generated from individual samples were assembled on the basis of the overlapping sequences (approximately 200 nts), to reconstruct complete genomes. Integrity of the ORFs in the genome sequences were checked using the NCBI ORFfinder algorithm (www.ncbi.nlm.nih.gov/orffinder/) and the Fangorn-Forest method implemented in the Geminivirus Data warehouse server (http://geminivirus.org:8080/geminivirusdw/)^37^. Sequences were then BLAST^38^ searched to identify and retrieve homologous sequences submitted in the GenBank. Additional GenBank sequences submitted from India and neighbouring countries were retrieved using the Geminivirus Data warehouse, or using accession numbers provided in the relevant recent literature^1,5,9,16,37^. To save computational time, redundant sequences were sorted out by running the datasets through the ElimDupes program implemented in the Los Alamos HCV sequence database server^39^ (https://hcv.lanl.gov/content/sequence/ELIMDUPES/elimdupes.html) and sequences representing a group of identical or near identical sequences (percent nucleotide identity, PNI ≥ 99%) were selected for further analysis.

Sequence datasets were aligned using the MUltiple Sequence Comparison by Log- Expectation algorithm (MUSCLE)^40^ (http://www.ebi.ac.uk/Tools/msa/muscle/), followed by molecular evolutionary analysis using MEGA^41^ version 6.06. Evolutionary distances were calculated employing the Maximum Composite Likelihood method and phylogenetic relatedness among the sequences were inferred using the Neighbour-Joining (NJ) algorithm. Reliability of the phylogenetic relatedness of the branches was evaluated by percentages obtained through 1000 bootstrap iteration of the datasets. Ambiguous nucleotides, gaps and missing data were not considered in analysis. Pairwise sequence comparisons were performed using Sequence Demarcation Tool (SDT)^42^, version 1.2.

Since geminiviruses undergo evolution through frequent recombinations^43^, ambiguities in sequence relatedness are common in conventional phylogenies. To prevent such ambiguities and to test reticulate evolution (signifying recombinations), we reconstructed Neighbour Network (NN), using the SplitsTree^44^ version 4.14.1, with Kimura-2 parameter distance correction, exclusion of gaps and parsimony uninformative sites. Presence of reticulation in NN has been shown to signify recombination and such analyses are shown to estimate tree topologies more accurately in datasets with recombinant sequences^45^.

To examine the recombination events and probable breakpoints, aligned datasets were analyzed through a set of algorithms, namely RDP, GENECONV, BOOTSCAN, MAXCHI, CHIMAERA, SISCAN, 3SEQ and PHYLPRO, incorporated in the RDP suite version 4.85, using default settings and standard *Bonferroni* correction^46^. Recombination events detected by at least three or more different algorithms with sufficient statistical support (P < 0.01) were considered as true events. Each of the events was further verified from breakpoint distribution plots and by comparing the UPGMA phylogenetic trees generated with genetic regions from major and minor parents.

### Diagnostic PCR for specific detection of CLCuKoV-Bu

To further verify the results obtained from complete genome analysis of the virus from the representative samples, all the samples collected from the fields were subjected to diagnostic PCRs for CLCuMuV and CLCuKoV-Bu. In brief, RCA enriched templates were separately amplified with primer pair CLCuV-1F/ CLCuV-4R (designed on alignment of CLCuMuV sequences obtained in the present study along with GenBank reference sequences) and primer pair CLCuKoV-Bu-F/ CLCuKoV-Bu-R, which was recently designed by Brown *et al*.^8^ for diagnostic detection of CLCuKoV-Bu. Each sample was run in duplicate.

### Data availability

Sequences generated in this study are available in the GenBank under the accession numbers KY120359 - KY120362, KY081413- KY081416, KY305676, MF929022-MF929036.

## Results

### Amplification and sequencing of begomovirus and satellite genomes

Initially, we attempted to screen the DNA extracts with universal primers for geminiviruses and satellites using direct PCR approach. Though we could get amplicons of expected size in most of the samples with DNA-A, alphasatellite and betasatellite primer pairs, but amplification was sub-optimal, along with presence of non-specific amplicons. To improve specific amplification, we adopted the RCA mediated circular ssDNA enrichment strategy for viral and satellite genetic components present in the total DNA extracts. In post-RCA PCR, we obtained optimal amplification in all the test DNA extracts with primer pairs for DNA-A, alphasatellite and betasatellite. In addition to amplicons of expected size, some subgenomic amplicons (approximately 0.7 kb and 1.1 kb) were also generated in PCR with betasatellite primers. However, even after repeated attempts we could not amplify DNA-B. Amplification results thus implicated monopartite begomovirus disease complex in our samples. Consequent to amplification, cloning, sequencing and assembling of the amplicons, 32 complete genome sequences each for DNA-A and betasatellite and 30 complete genome sequences for alphasatellite were available for further analyses.

### Diversity of the sequences and results of BLAST analysis

Sequences were initially screened through the ElimDupes program to identify identical or near identical sequences (≥99% cut-off). Begomovirus sequences were found to segregate into four groups, and one sequence representing each of these four groups was selected for further analyses and submitted to the GenBank (isolates S78-S81; GenBank accession numbers KY120359- KY120362). Similarly, five representative betasatellite sequences were selected for subsequent analyses (isolates S82-S85 and S90; GenBank accession numbers KY081413- KY081416 and KY305676). Detailed features of the representative virus and betasatellite sequences are presented in Table 1. Considering the higher diversity of alphasatellite genome in the present study, 14 representative sequences were analyzed in details (isolates C31, C35-C40 and C43-C48, C51; GenBank accession numbers MF929022- MF929028, MF929030- MF929036). Detailed features of the representative alphasatellite can be found online as Supplementary Table S2.

**Table 1:** Detailed features of the complete DNA-A and betasatellite sequences generated in the present study.

Begomovirus										
Isolate No. (GenBank Accession No.)	Length (nts)	Number (%) of similar clones	Begomovirus ORFs (position/ nts/aa)							Betasatellite ORF (position/ nts/aa)
			Coat Protein (AV1)	pre-coat protein (AV2)	replication associated protein (Rep, AC1)	transcription activator protein (TrAP, AC2)	replication enhancer protein, (REn, AC3)	regulatory protein (AC4)	AC5 (uncharacterized Protein)	βC1 (beta C1 protein)
S78 (KY120359)	2738	7 (22%)	276–1046/ 771/256	116–472/ 357/118	2583–2185/ 399/132* (defective)	1598–1146/ 453/ 150	1453–1049/ 405/134	2429–2127/ 303/100	791–60/732/243	—
S79 (KY120360)	2738	12 (38%)	276–1046/771/256	116–472/357/118	2583–1495/ 1089/362	1598–1146/ 453/ 150	1453–1049/ 405/134	2429–2127/ 303/100	791–60/732/243	—
S80 (KY120361)	2738	10 (31%)	276–1046/771/256	116–472/357/118	2583–1495/ 1089/362	1598–1146/ 453/ 150	1453–1049/ 405/134	2429–2127/ 303/100	791–60/732/243	—
S81(KY120362)	2750	3 (9%)	276–1046/771/256	116–472/357/118	2673–1495/ 1179/392	1598–1146/ 453/ 150	1453–1049/ 405/134	2429–2127/ 303/100	941–60/882/293	—
Betasatellite										
S83 (KY081413)	1361	14 (44%)	—	—	—	—	—	—	—	551–195/357/118
S84(KY081414)	1378	9 (28%)	—	—	—	—	—	—	—	551–195/357/118
S85 (KY081415)	1364	5(16%)	—	—	—	—	—	—	—	551–195/ 357/118
S82 (KY081416)	688	2 (16%)	—	—	—	—	—	—	—	defective
S90 (KY305676)	1171	2 (9%)	—	—	—	—	—	—	—	444–83 (partial βC1 like)

*Premature Stop codon due to UAC to UGA at codon position 133.

All the DNA-A sequences had a length of approximately 2.7 kb and a genetic arrangement and ORFs, typical for begomoviruses. Among the DNA-A genomes, sequences KY120359, KY120360 and KY120361 were similar and shared PNI score of 98.1% ± 0.6 (mean ± SD) among themselves. However, these three sequences had a significantly less PNI score (93.0% ± 1.0, P < 0.001) when compared to the sequence KY120362. The difference among the sequences (KY120356 - KY120361 vs. KY120362) was also evident in the BLASTn homology search (megablast). Sequences KY120359, KY120360 and KY120361 were primarily found to be most homologous (94–97% identity at 98–100% query coverage) to three GenBank CLCuMuV sequences isolated from Vehari, Pakistan^1^ during 2015 (KX656810, KX656806, KX656809) followed by other sequences isolated from different parts of northwestern India during 2012–14 (KJ959628, KX831888, KM096467, KM096469, KX831891 etc.). On the other hand, sequence KY120362 showed best match (maximum 94% identity at 100% query coverage), with CLCuMuV sequences recently reported from northern and northwestern India (KY561820, KX951461, KX951460, KJ868820, JN678804) followed by sequence from Pakistan (EU384573, AJ002447, AJ002458, AJ496287). Additionally, SDT analysis of the present samples with relevant NCBI RefSeq sequences and type sequences^5^ of each of the CABs and their respective strains demonstrated the presence of two different strains of CLCuMuV in the infected samples (Supplementary Fig. S3).

On the other hand, betasatellite sequences of various lengths including subgenomic sequences with defective or intact coding regions were detected in the present samples. Of the 5 representative sequences, KY081413 - KY081415 denoted typically complete sequences with varying lengths (1361–1378 nts) and with intact βC1 coding region. These sequences shared PNI of 88–95% among themselves and showed close homology (up to 100% query coverage and up to 92–99% identity) with a number of GenBank CLCuMuB sequences, recently isolated from India and Pakistan. However, sequences KY081416 (688 nts) and KY305676 (1171 nts) denoted subgenomic betasatellite sequences and in BLAST analysis, showed maximum homology (94–98% identity) with defective interfering or subgenomic CLCuMuB sequences, recently isolated from Rajasthan and Punjab of northwestern India. SDT analyses results of betasatellite sequences along with NCBI RefSeq betasatellite sequences can be found online as Supplementary Fig. S4.

Interestingly, as compared to the DNA-A and betasatellite sequences, genetic diversity of alphasatellite was found to be considerably high in the present samples. Of the 30 complete alphasatellite sequences, based on the results of ElimDupes program (≥99% cut-off), 14 representative sequences were analyzed for further analyses. Although these sequences have PNI ranging from 67–98% among themselves and varied in length (ranging from 1362–1378 nts), but all the sequences retained an intact alpha Replication initiator protein (Rep) ORF of identical length and an A-rich region of variable length. On BLASTn analysis, these sequences were found to be homologous (100% query coverage and 93–99% identity) to recent GenBank alphasatellite sequences isolated primarily from Pakistan and belonged to four different species, namely, *Okra leaf curl alphasatellite* (OLCuA), *Gossypium darwinii symptomless alphasatellite* (GDarSLA), Ageratum *yellow vein India alphasatellite* (AYVIA) and *Tomato leaf curl alphasatellite* (ToLCA). Of these ToLCA was the most frequently encountered species within the clonal sequences followed by OLCuA, GDarSLA and AYVIA. Details of alphasatellite sequence BLAST analyses results can be found online as Supplementary Table S2.

### Phylogenetic analysis of the begomovirus DNA-A and satellite sequences

**Figure 2:**
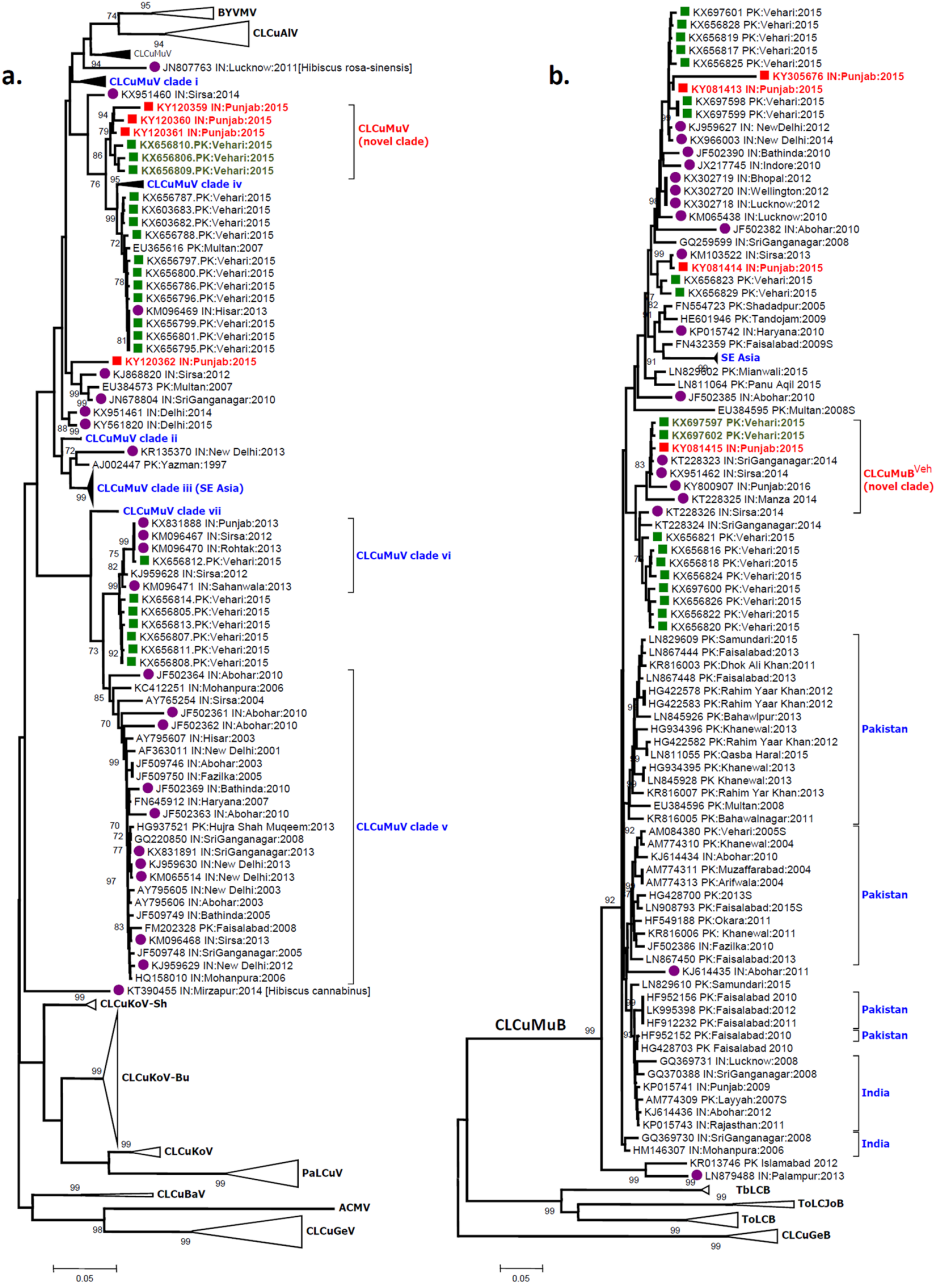
Neighbour-Joining (NJ) phylogenetic trees reconstructed for complete genome DNA-A (panel a) and betasatellite (panel b) sequences generated in the present study along with GenBank sequences. Sequences generated in the present study (Punjab, India, 2015) are represented by red solid squares and red font, sequences recently isolated from Vehari, Pakistan (2015)^1^ are represented by solid green squares and sequences isolated from other parts of northwestern India during previous few years (2010 onwards) are represented by solid purple rounds. CLCuMuV clades demarcated in the panel A are according to a recent paper^9^. Begomovirus species names have been abbreviated according to the recent standard nomenclature^5^. Novel clade of CLCuMuV, as proposed in the present work is marked (panel a). Novel clade of CLCuMuB^Veh^ as proposed recently by Zubair *et al*.^1^ is marked (panel b). Some branches in both the trees (not relevant in the current perspective) have been collapsed for better representation of the relevant information.

In the NJ phylogenetic tree reconstructed with 244 GenBank DNA-A sequences along with the present sequences (Fig. 2a), the reference sequences segregated according to the clades described recently for CLCuMuV^9^. Corroborating with the BLASTn results, sequences generated in this study were found to be phylogenetically affiliated to CLCuMuV, of which sequences KY120359, KY120360 and KY120361 together with three recent sequences isolated from Vehari, Pakistan^1^ (KX656806, KX656809 & KX656810) formed a distinct clade, supported by high bootstrap value (Fig. 2a). This clade further bifurcated into two subclades, differentiating the present sequences from that of Pakistan, signifying their origin from a common progenitor, and their divergence subsequently. This clade was evidently distinct from all the seven other previously reported clades of CLCuMuV sequences^9^, which indicates recent emergence of these sequences. Phylogenetically, this clade was most closely related to the CLCuMuV clade iv, which is represented by sequences originating mostly from India (Fig. 2a). Interestingly, the three Pakistan sequences (KX656806, KX656809 & KX656810)^1^ that composed the new clade along with our sequences, were found to be phylogenetically divergent from other CLCuMuV sequences isolated from the same site, during the same study. On the other hand, sequence KY120362 separately clustered with sequences belonging to CLCuMuV, isolated from India (JN678804 & KJ868820) and Pakistan (EU384573).

**Figure 3:**
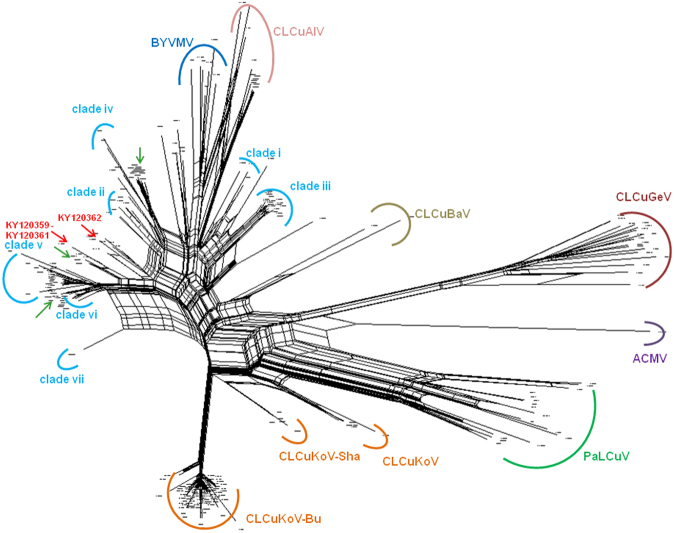
Neighbour Net analysis of CLCuD associated begomoviruses generated in the present study along with GenBank sequences. Extensive network structures among the CLCuMuV sequences indicate considerably conflicting phylogenetic signals, suggesting widespread recombination within the sequences. CLCuMuV clade demarcations are according to a recent paper^9^. Begomovirus species names have been abbreviated according to the recent standard nomenclature^5^. Locations of the sequences generated in the present study (Punjab, India, 2015) are indicated by red arrowheads, while those isolated very recently from Vehari, Pakistan (2015)^1^ are indicated by green arrowheads.

In NN analysis (Fig. 3), agglomeration of the different clades of CLCuMuV was observed to be analogous to the NJ tree (Fig. 2a). The NN tree demonstrated extensive reticulation among the different clades of CLCuMuV, signifying frequent inter and intragenic recombinations. Nevertheless, in the NN tree also, our sequences clustered with three particular sequences (KX656806, KX656809 & KX656810) from Vehari, Pakistan^1^, and collectively symbolize the emergence of a separate lineage. The basal network of this CLCuMuV lineage appears to indicate its origin from a recombinant ancestor, phylogenetically interspersed between CLCuMuV clades (ii), (iv), (v) and (vi), indicating the contribution of these clades in emergence of the present sequences. An interesting observation of the NN tree was the extremely high reticulation among the CLCuMuV sequences, as compared to other CABs, suggesting relatively higher incidences of genetic recombination occurring among CLCuMuV and its strains.

On the other hand, in the phylogenetic tree generated with CLCuMuB sequences, sequences previously isolated (before 2013) from India and Pakistan appeared to form distinct clusters, although there were a few sequences that clustered irrespective of the country of origin (Fig. 2b). CLCuMuB sequences from Southeast Asian countries were also found to cluster separately, indicating their monophyletic origin. Very interestingly CLCuMuB sequences generated in the present study clustered with a number of CLCuMuB sequences recently isolated from Vehari, Pakistan^1^, along with sequences very recently (after 2013) isolated from other places of northwestern India and Pakistan (Fig. 2b). Particularly one of the present sequences (KY081415) along with a number of betasatellite sequences very recently isolated from different parts of northwestern India (KT228323, KX951462, KY800907, KT228325) were found to cluster separately with the recently denoted CLCuMuB^Veh^ sequences (KX697597 & KX697602)^1^. The observed phylogenetic relatedness of the present sequences, their divergence from previously circulating betasatellite sequences in this region indicates a relatively recent emergence of the present betasatellites sequences, corroborating with the data obtained from DNA-A sequences.

**Figure 4:**
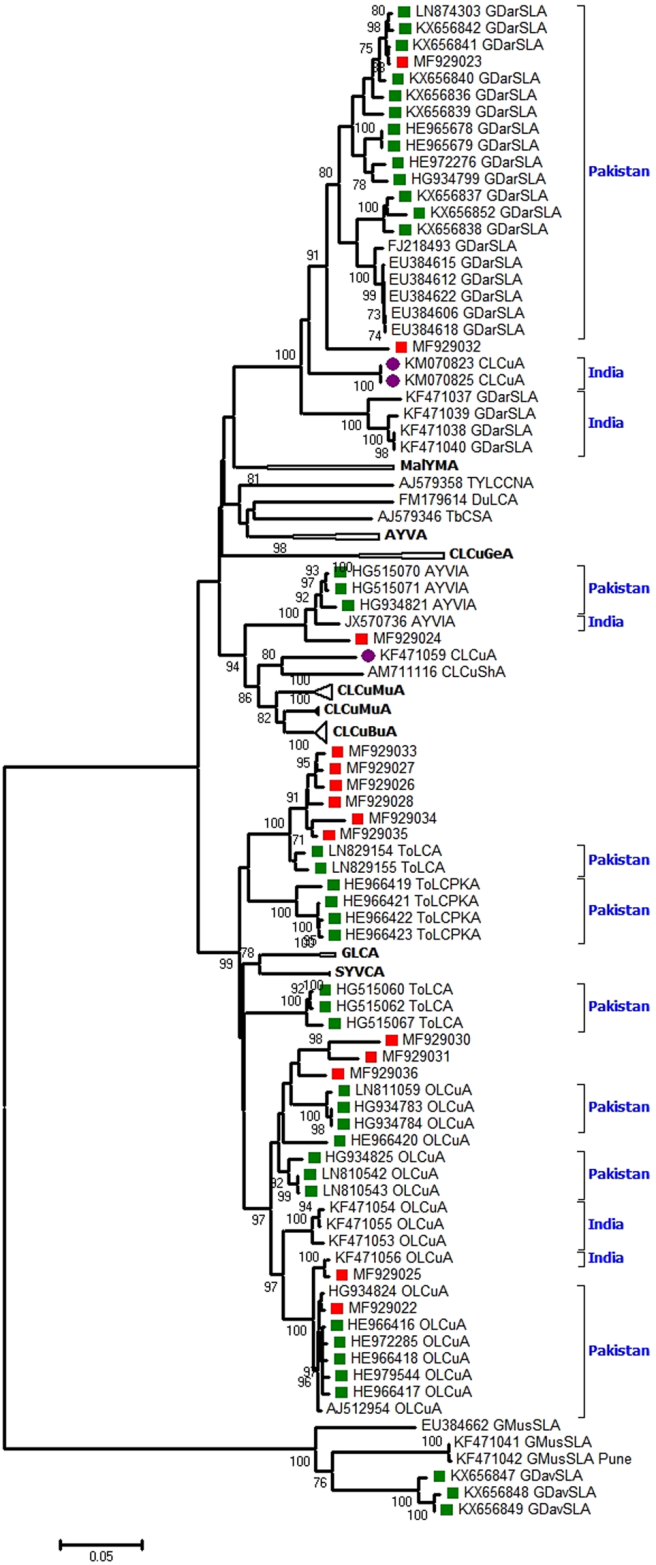
Neighbour-Joining (NJ) phylogenetic tree reconstructed for complete genome alphasatellite sequences generated in the present study along with GenBank sequences. Sequences generated in the present study (Punjab, India, 2015) are represented by red solid squares, sequences recently (2011 onwards) isolated from Pakistan are represented by solid green squares while sequences from different parts of northwestern India are represented by solid purple rounds. Species names have been abbreviated according to the standard ICTV nomenclature for alphasatellites. Some branches in the tree (not relevant in the current perspective) have been collapsed for better representation of the relevant information.

In phylogenetic tree generated with reference GenBank alphasatellite sequences and BLASTn returned homologous sequences, clustering of present sequences was in accordance with the BLASTn results. Interestingly, one of the GDarSLA sequence (MF929023) isolated in the present study was observed to be phylogenetically related to alphasatellite sequences (KX656836, KX656839-KX656842) recently reported in cotton from Pakistan^1^ (Fig. 4). Similarly all the ToLCA sequences also clustered with two recent ToLCA sequences from Pakistan (LN829154, LN829155). On the other hand, AYVIA and OLCuA sequences were also found to be phylogenetically affiliated to sequences mainly from Pakistan, along with some Indian isolates.

### Analyses of recombination in DNA-A and satellite sequences

**Figure 5:**
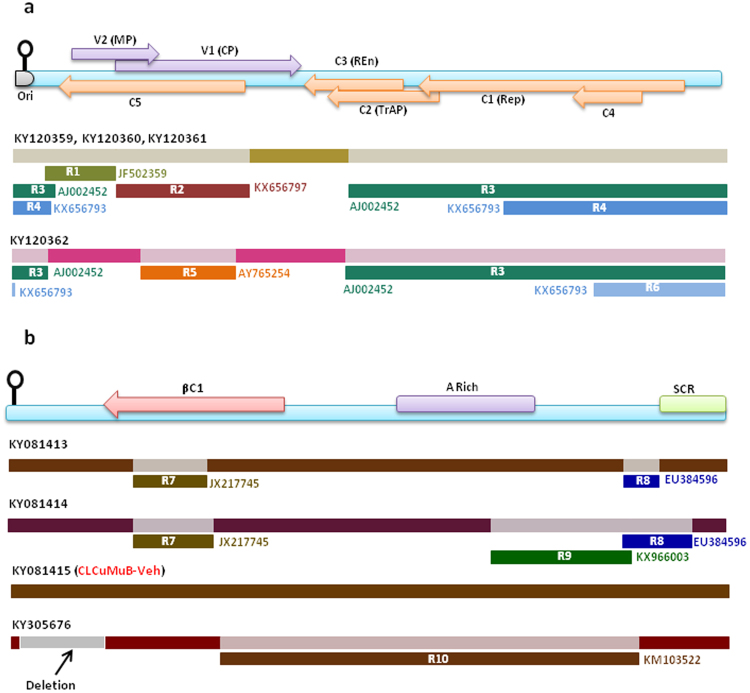
Recombination patterns observed in the complete genome DNA-A (panel a) and betasatellite (panel b) obtained in the present study. Genetic map of DNA-A and betasatellite is shown at the top of the respective panels. Recombinant fragments are represented by different colour bars along with the GenBank accession numbers of the minor parent involved in each recombination event. Due to similarity, recombination events observed in three of the present sequences (KY120359-KY120361) have been represented in a single graphical. Different recombination events in the DNA-A are denoted by R1-R6, while those in betasatellite are denoted by R7-R10. Details of these recombination events can be found in Table 2 (DNA-A) and Table 3 (betasatellite).

Analysis of the present DNA-A sequences showed clear events of inter-species and intra-species recombinations involving most of the coding regions and strongly supported by statistical signature. Details of the recombination events are summarized in Fig. 5a and Table 2. Specifically, two different patterns of recombination namely R1 (between two different CLCuMuV sequences) and R2 (Between CLCuMuV and CLCuKoV) were common in the three representative sequences analysed here (KY120359, KY120360 and KY120361). On the other hand, events R3 and R4 signify CLCuAlV and CLCuKoV as parental sequence and were common to all the four representative sequences (Table 2). Except the relative alteration in the site of event R4 in KY120361, probable breakpoints and span of the recombinant fragment was similar in all the events, suggesting a common origin for the sequences. Apart from the above four events, sequence KY120362 had an additional recombinant (R5) fragment (CLCuMuV and CLCuKoV). Among these events, individual recombinant fragment or a combination of fragments were observed in a number of GenBank sequences previously isolated from India and Pakistan. Strongly corroborating with the BLASTn and phylogenetic analyses, sequences recently isolated from Vehari, Pakistan^1^ (particularly KX656806, KX656809 and KX656810) had precisely similar combination of events (Supplementary Fig. S5). Moreover, in two of the events, namely R2 and R4, sequences recently isolated from Vehari, Pakistan served as parents, signifying the epidemiological relationship among the Pakistan sequences with ours. Recombination event R5 also signified recombination involving CLCuMuV and CLCuKoV-Bu sequences from India and Pakistan respectively.

**Table 2:** Statistical features of recombination events observed in complete DNA-A sequence analyzed in the present study.

Recombination Event	Probable Breakpoints		Recombinant Sequence(s)	Parental Sequence(s)		P-value calculated by different recombination detection algorithms						
	Begin	End		Minor	Major	RDP	GENECONV	Bootscan	Maxchi	Chimaera	SiSscan	3Seq
R1	47	326	KY120359 KY120360 KY120361	JF502359 (CLCuMuV-Ra) SriGanganagar, India, 2005	EU384574 (CLCuMuV-Ra) Multan, Pakistan, 2007	8.32E-14	9.39E-11	1.58E-12	1.60E-07	5.54E-08	1.83E-48	1.98E-10
R2	328	887	KY120359 KY120360 KY120361	KX656797 (CLCuMuV) Vehari, Pakistan, 2015	AJ002448 (CLCuKoV) Faisalabad, Pakistan, 1996	9.17E-58	5.32E-54	3.05E-49	1.30E-20	1.27E-20	2.21E-24	9.92E-11
R3	1300	79	KY120359 KY120360 KY120361 KY120362	AJ002452 (CLCuAaV) Alabad, Pakistan, 1996	HE985227 (CLCuKoV-Bu) Okara, Pakistan, 2012	3.45E-39	3.64E-45	9.30E-41	3.84E-30	5.81E-23	2.32E-46	1.68E-27
R4/R6	1962/ 2329*	65/7*	KY120359 KY120360 KY120361 KY120362	KX656793 (CLCuAlV) Vehari, Pakistan, 2015	JF510458 (CLCuKoV-Bu) Bathinda, India, 2010	1.06E-68	7.05E-62	9.18E-67	1.84E-31	1.16E-32	3.42E-36	8.64E-64
R5	443	834	KY120362	AY765254 (CLCuMuV-Ra) Sirsa, India, 2004	HE985227 (CLCuKoV) Okara, Pakistan, 2012	1.50E-45	2.92E-44	9.05E-36	1.45E-16	1.41E-16	2.94E-25	9.92E-11

*For recombination event R6.

Among the betasatellite sequences generated in this study, four different recombination events were detected. Details of the recombination events are presented in Fig. 5b and Table 3. Events R7 (within the βC1 coding region), R9 and R10 involved CLCuMuB sequences from India, while event R8 and R9 involved *Tomato leaf curl Joydebpur betasatellite* (ToLCJoB) and *Tobacco leaf curl betasatellite* (TbLCuB) sequences respectively as minor parents, and included the A-rich and SCR regions.

**Table 3:** Statistical features of recombination events observed in complete DNA-β sequence analyzed in the present study.

Recombination Event	Probable Breakpoints		Recombinant Sequence(s)	Parental Sequence(s)		P-value calculated by different recombination detection algorithms						
	Begin	End		Minor	Major	RDP	GENECONV	Bootscan	Maxchi	Chimaera	SiSscan	3Seq
R7	228	448/472*	KY081413 KY081414	JX217745 (CLCuMuB) Indore, India, 2010	KY800907 (CLCuMuB) Punjab, India, 2016	3.06E-04	2.64E-02	NS	NS	NS	NS	1.40E-02
R8	1164	1259/1316*	KY081413 KY081414	EU384596 (CLCuMuB) Multan, Pakistan, 2008	AJ966244 (ToLCJoB) Gazipur, Bangladesh, 2003	1.38E-24	6.21E-26	4.16E-19	3.03E-05	1.14E-04	6.24E-06	2.02E-11
R9	945	1189	KY081414	KX966003 (CLCuMuB) New Delhi, India, 2014	HG983281 (TbLCuB) Yazman, Pakistan, 2013	3.57E-07	6.27E-08	1.36E-07	NS	NS	1.10E-13	NS
R10	375	1095	KY305676	KM103522 (CLCuMuB) Sirsa, India, 2013	KY081413 (CLCuMuB) Punjab, India, 2015	2.08E-07	5.98E-10	1.24E-06	3.45E-02	3.19E-02	1.35E-15	1.81E-05

*For KY081414.

Similarly, frequent recombination was also observed within the alphasatellite sequences. Evidences of recombinants spanned across the alpha Rep ORF as well as the A-rich region. Our alphasatellite sequences belonging to different species had evidences of GDarSLA, CLCuMuA, CLCuBuA and OLCuA sequences. Most diverse patterns of recombinations were observed within the OLCuA sequences. ToLCA sequences were quite similar to each other in terms of parental sequences, and differed only in the span of recombinant fragment, suggesting a common source of origin. Recombination pattern of the GDarSLA sequences were found to be matching to that of GDarSLA sequences (KX656836, KX656840-KX656842) recently reported from Paksitan^1^. Details of the recombination patterns and statistical features observed in the present alphasatellite sequences can be found online as Supplementary Fig. S6 and Table S3.

### Results of diagnostic PCR for CLCuKoV-Bu

Using diagnostic PCR with primers specific for CLCuKoV-Bu, we could not detect amplicons in any of the sample extracts, collected during the present study. However, amplicons were clearly detectable in PCR tubes with primers designed for amplifying CLCuMuV sequences.

## Discussion

Taken together, results of this study demonstrated the exclusive detection of CLCuMuV in plant samples collected from the most recent CLCuD outbreak in Punjab (India). However, we could not detect CLCuKoV-Bu sequences in any of the present samples, although we have used a highly sensitive RCA-PCR based clonal sequencing approach and specific diagnostic PCR assay. This finding was striking, since almost a decade CLCuKoV-Bu has prevailed in the Indian subcontinent and has also been associated with a number of severe outbreaks of CLCuD in India and Pakistan, until recently^1,3,6,8,9,16^. Our results thus connote the rebound of CLCuMuV in this region of the Indian subcontinent, signifying a shifting epidemiology of CLCuD.

Our findings of the rebound of CLCuMuV in Punjab, India corroborates with the recent findings of Zubair *et al*.^1^, documenting the return of CLCuMuV in CLCuD affected cotton plants in Vehari, Multan, Pakistan^1^. Comparative analyses of virus and satellite sequences from both these studies revealed a strong phylogenetic relatedness among them. Furthermore, these recent sequences were clearly distinct from sequences previously isolated from the Indian subcontinent. Recombination patterns of the CLCuMuV and a number of associated satellite sequences from both these studies were also precisely similar and were unique, as compared to previously reported sequences. These evidences suggest towards a strong epidemiological connection among the CAB complexes reported in both these studies. Another significant finding of both these studies was the complete non-detection of CLCuKoV-Bu sequences. This finding was quite surprising, since the study by Zubair *et al*.^1^ was done on samples from Vehari, Pakistan, where the CLCuKoV-Bu was first reported^18^. Collectively, these observations provide molecular evidences signifying the re-emergence of CLCuMuV in the Punjab region (of both India and Pakistan), which in turn imply a shift in molecular epidemiology of the predominant CABs in the Indian subcontinent.

Apart from the similarities mentioned above, our findings differed from that of Zubair *et al*.^1^, in certain aspects. Of these, re-appearance of CLCuKoV and CLCuAlV, along with CLCuMuV in Pakistan^1^ is interesting. Although presence of recombination fragments from these two species was indicated in one of our present sequences (KY120362) but we could not detect these two CAB species *per se*, in our samples. Notably CLCuKoV and CLCuAlV are believed to have emerged in Pakistan, but as compared to the widely distributed CLCuMuV and CLCuKoV-Bu (these two also have evolved in Pakistan), CLCuKoV and CLCuAlV are sporadically detected in India^16,21^. We assume that this differential distribution of CABs might be attributable to the transmission efficiency of the whitefly species prevalent in the Indian subcontinent, since it has recently been demonstrated that transmission efficiency for a given begomoviruses varies significantly with the species of the whitefly^47^. It therefore remains an exciting topic for further research to examine the transmission efficiency of different CABs by Asia II-1 species of *B*. *tabaci*, which is the single predominant whitefly species in this part of the Indian subcontinent^2,3^.

On the other hand, alphasatellites ToLCA and OLCuA were frequently detected in our study samples, contrasting the detection of GDarSLA, GDavSLA and CLCuBuA by Zubair *et al*.^1^. CLCuBuA and CLCuMuA specific fragments were detected only as contributing parents in the ToLCA and OLCuA sequences analyzed in this study. Interestingly, all the present ToLCA sequences were found to be homologous (95–98% match over 100% query coverage) and phylogenetically related to two GenBank sequences (LN829154 and LN829155), very recently isolated from cotton from Pakistan. Similarly, most of the OLCuA sequences determined in this study were phylogenetically more related to OLCuA sequences recently reported from Pakistan^48^, as compared to OLCuA sequences recently isolated from northern India^49^. To the best of our knowledge, association of alphasatellites other than CLCuMuA and CLCuBuA with CABs has not been reported in previous CLCuD outbreaks. Altogether, the results of the alphasatellite sequence analyses also attest to the recent epidemiological linkage of different genetic components of the present begomovirus disease complex with those circulating in Pakistan.

Very recently, Sattar *et al*.^3^ analyzed the CLCuD incidence data from 1997–2015, and observed a radical decline in the incidence of the ongoing “*Burewala epidemic*” during 2014–15. They anticipated this phase as pre-epidemic virus incubation period for the next epidemic in this region. Further, they predicted the probability of involvement of CLCuKoV-Bu (with intact C2), CLCuMuB and ToLCNDV with the next epidemic (*third epidemic*) of CLCuD in the Indian subcontinent^3^. Based on statistical modelling of the climate data, they also predicted Multan, Pakistan (origin site of the *Multan* and *Burewala* epidemics) as the site of emergence of newer CABs and their subsequent spread across the borders^3^. Taking these predictions into consideration, it appears that the reappearance of the previous species/strains in Vehari, Pakistan^1^ and the detection of alike sequences with the recent CLCuD outbreak in Punjab, India hallmark the ‘*third epidemic’*, as predicted by Sattar *et al*.^3^. However, in contrast to the predictions by Sattar *et al*., non-detection of CLCuKoV-Bu and/or other bipartite virus in the present study or in the study by Zubair *et al*.^1^, we hypothesize that the re-emerging CLCuMuV complex or its descendants will be accountable for impending epidemics in the Indian subcontinent. Our assumptions also concur with the findings of Brown *et al*.^8^, demonstrating that certain core CABs, including CLCuMuV and CLCuKoV-Bu are endemic to the Indian subcontinent and are expected to prevail in this region. The study also established that as compared to others, certain CAB complexes undergo rapid genetic diversification, which may lead to expansion of their host range, transmission potentiality, virulence and resistance breaking capability^8^. From this perspective, high genetic diversity of CLCuMuV and satellite complexes as revealed in the present study seem to justify their evolution and re-emergence with novel recombinations.

Changing epidemiology of CLCuD in Pakistan side of the Indian subcontinent has been well documented in the recent literature^1,3^. In India too, indications were evident from the results of recent field trials of certain Bt cotton varieties conducted at various locations of northern and northwestern India^16,26,30^. During these trials, a number of cotton varieties that were resistant against CLCuD during crop season 2011–2012 suddenly showed susceptible or highly susceptible reaction during the subsequent crop seasons^26^. Notably, around 90% of cotton crops presently being cultivated in the Indian subcontinent are Bt cotton hybrids, which were primarily introduced to prevent the menace of bollworm complex^3,19,20,25,50,51^. However, a large number of these Bt cotton varieties are either susceptible to CLCuD or their tolerance limits have not been thoroughly scrutinized^25,26,51^. During the present outbreak, it was observed that Bt hybrid cotton varieties (*G*. *hirsutum*) were destroyed specifically, while indigenous cotton species (*G*. *arboreum*) remained unaffected, which has been observed during previous outbreaks too^19,21,25,26,50,51^. It has been hypothesized that *G*. *arboreum* being native to the Indian sub-continent, has developed immunity against the Asian virus complexes due to its long co-evolutionary history, while the varieties of the exotic *G*. *hirsutum*, which were introduced into the Indian subcontinent from Mexico have not developed resistance and are still susceptible to Asian CLCuVs^10,14,52^.

Interestingly, recent studies demonstrate that Bt hybrid cotton varieties are non-toxic to hemipteran pests (including whiteflies) and may perhaps augment their populations^53,54^. Additionally, indiscriminate use of pesticides over the years for pest control has induced exceptionally high magnitudes of pesticide resistance among the whitefly populations in the Indian subcontinent, which plays an important role in rapid dispersion of the virus complex causing outbreaks^2,3,25,55^. Nevertheless, widespread adoption of Bt cotton has dramatically changed the pest complex scenario in this cotton ecosystem, signifying a drastic decline in bollworms, while encouraging the emergence of several sap feeding Hemipterans, including whiteflies, aphids, thrips etc. as serious pests, in the recent years^56^. Therefore, immediate re-evaluation of culture and control practices is required for management of Bt- cotton, where threat of CABs is significantly greater as compared to bollworms.

India and Pakistan rank third and fourth respectively from the perspective of global cotton production (FAOStat, 2013)^57^. Economies of both these countries greatly depend on cotton production. Due to the geographical immediacy of India and Pakistan, viral species and recombinants evolving in one of these regions spread rapidly to the other^1,3,6,9,10^. Although CAB complexes circulating in Pakistan are well characterized, there is a scarcity of scientific literature on the genetic diversity of CAB complexes in the Indian side. To the best of our knowledge, this is the first study from India to report in-depth genetic characterization of the CAB complex directly isolated from outbreak stricken fields. Overall, results of this study correlate with recent literature and field studies, signifying a changing epidemiology of CLCuD in this region.

In conclusion, our results strongly emphasize the necessity of stringent and regular monitoring of the prevailing CAB complexes in this region. This will definitely help in early detection of emerging or re-emerging etiological viral agents, thereby averting future outbreaks. Apart from the viruses, it is also equally important to rigorously screen the cotton varieties, especially the Bt cotton varieties, targeted for cultivation in a given region, against CABs prevalent in that region. Varieties found to be resistant in one geographical region should not be implied in other regions and rigorous field trials under natural conditions must be performed before their release for mass cultivation. In addition, resistant nature of the released varieties needs to be validated more often to identify any sudden change in susceptibility and to correlate such events with the molecular epidemiology of the CAB complexes. In parallel, cultivation of the indigenous cotton varieties should be encouraged, since these varieties have repeatedly been seen to be highly resistant to CLCuD in this region. Finally, there is an urgent need to revisit, reformulate and enforce sustainable agriculture trade practices, to prevent outbreaks of this socio-economically devastating disease in future.

We wish to dedicate this research to the everlasting memory of our colleague and co-author of this work, *Raghvendra Budhauliya*, whose untimely departure is an irrevocable loss to us all.

## Electronic Supplementary material

Supplementary Information 

## References

[1] Zubair M (2017). Multiple begomoviruses found associated with cotton leaf curl disease in Pakistan in early 1990 are back in cultivated cotton. Sci. Rep..

[2] Naveen NC (2017). Insecticide resistance status in the whitefly, *Bemisia tabaci* genetic groups Asia-I, Asia-II-1 and Asia-II-7 on the Indian subcontinent. Sci. Rep..

[3] Sattar MN, Iqbal Z, Tahir MN, Ullah S (2017). The Prediction of a New CLCuD Epidemic in the Old World. Front. Microbiol..

[4] Zerbini FM (2017). ICTV Virus Taxonomy Profile: Geminiviridae. J. Gen. Virol..

[5] Brown JK (2015). Revision of Begomovirus taxonomy based on pairwise sequence comparisons. Arch. Virol..

[6] Sattar MN, Kvarnheden A, Saeed M, Briddon RW (2013). Cotton leaf curl disease–an emerging threat to cotton production worldwide. J. Gen. Virol..

[7] Briddon RW, Stanley J (2006). Subviral agents associated with plant single-stranded DNA viruses. Virology..

[8] Brown JK (2017). Molecular diagnostic development for begomovirus-betasatellite complexes undergoing diversification: A case study. Virus. Res.

[9] Saleem H (2016). Diversity, mutation and recombination analysis of cotton leaf curl Geminiviruses. PLoS One..

[10] Nawaz-ul-Rehman MS, Briddon RW, Fauquet CM (2012). A melting pot of Old World begomoviruses and their satellites infecting a collection of Gossypium species in Pakistan. PLoS. One..

[11] Amrao L (2010). Cotton leaf curl disease in Sindh province of Pakistan is associated with recombinant begomovirus components. Virus. Res..

[12] Idris AM, Briddon RW, Bull SE, Brown JK (2005). Cotton leaf curl Gezira virus satellite DNAs represent a divergent, geographically isolated Nile Basin lineage: predictive identification of a satDNA REP-binding motif. Virus. Res..

[13] Leke WN, Khatabi B, Mignouna DB, Brown JK, Fondong VN (2016). Complete genome sequence of a new bipartite begomovirus infecting cotton in the Republic of Benin in West Africa. Arch. Virol..

[14] Jones RAC (2009). Plant virus emergence and evolution: Origins, new encounter scenarios, factors driving emergence, effects of changing world conditions, and prospects for control. Virus. Res..

[15] Ha C (2008). Molecular characterization of begomoviruses and DNA satellites from Vietnam: additional evidence that the New World Geminiviruses were present in the Old World prior to continental separation. J. Gen. Virol..

[16] Godara S, Paul Khurana SM, Biswas KK (2017). Three variants of cotton leaf curl begomoviruses with their satellite molecules are associated with cotton leaf curl disease aggravation in New Delhi. J. Plant Biochem. Biotechnol..

[17] Sohrab SS, Azhar EI, Kamal MA, Bhattacharya PS, Rana D (2014). Genetic variability of Cotton leaf curl betasatellite in Northern India. Saudi. J. Biol. Sci..

[18] Mansoor S (2003). Breakdown of resistance in cotton to cotton leaf curl disease in Pakistan. Plant. Pathol..

[19] Monga D, Manocha V, Chandkumhar K, Seni K, Pal Singh N (2011). Occurrence and prediction of cotton leaf curl virus disease in northern zone. J. Cotton Res. Dev..

[20] Monga, D., Chakrabarty, P. K. & Kranthi, R. Cotton leaf Curl Disease in India-recent status and management strategies. *5th meeting of Asian Cotton Research and Development Network* http://www.icac.org/tis/regional_networks/asian_network/meeting_5/documents/papers/PapMongaD.pdf (2011).

[21] Rajagopalan PA (2012). Dominance of resistance-breaking cotton leaf curl Burewala virus (CLCuBuV) in northwestern India. Arch. Virol..

[22] Varma, A. *et al*. Leaf curl disease of cotton in North-West-India. Report of the ICAR Committee (1995).

[23] Rishi N, Chauhan MS (1994). Appearances of leaf curl disease of cotton in Northern India. J. Cotton. Res. Dev..

[24] Zaffalon V, Mukherje eSK, Reddy VS, Thompson JR, Tepfer M (2012). A survey of Geminiviruses and associated satellite DNAs in the cotton-growing areas of northwestern India. Arch. Virol..

[25] Kranthi, K. R. Whitefly –The Black Story. *Cotton Statistics & News*, http://cicr.org.in/pdf/Kranthi_art/Whitefly.pdf (2015).

[26] Indian Council of Agricultural Research (ICAR)- All India Coordinated Research Project on Cotton – Annual Report (2015–16) http://www.aiccip.cicr.org.in/CD_15_16/contents.htm (2016).

[27] Cotton Corporation of India. Statistics of state wise cotton area, production and productivity. http://cotcorp.gov.in/statistics.aspx?pageid=4#area1 (2016).

[28] Varma, S. & Bhattacharya, A. Whitefly destroys 2/3rd of Punjab’s cotton crop, 15 farmers commit suicide. *The Times of India* http://timesofindia.indiatimes.com/india/Whitefly-destroys-2/3rd-of-Punjabs-cotton-crop-15-farmers-commit-suicide/articleshow/49265083.cms (2015).

[29] Vasudeva, V. GM cotton: whitefly attack raises anxiety among farmers. *The Hindu* http://www.thehindu.com/sci-tech/agriculture/gm-cotton-whitefly-attack-raises-anxiety-among-farmers/article7775306.ece (2015).

[30] Godara S, Saini N, Paul Khurana SM, Biswas KK (2015). Lack of resistance in cotton against cotton leaf curl begomovirus disease complex and occurrence of natural virus sequence variants. Indian Phytopath..

[31] Datta S (2015). First report of Tomato leaf curl Bangladesh virus (ToLCBV) infecting *Gomphostemma niveum* plants in Assam, India. New Dis Rep..

[32] Khan ZA, Abdin MZ, Khan JA (2015). Functional Characterization of a Strong Bi-directional Constitutive Plant Promoter Isolated from Cotton Leaf Curl Burewala Virus. PLoS One..

[33] Rojas MR, Gilbertson RL, Russell DR, Maxwell DP (1993). Use of degenerate primers in the polymerase chain reaction to detect whitefly-transmitted Geminiviruses. Plant. Dis..

[34] Chowda-Reddy RV, Colvin J, Muniyappa V, Seal SE (2005). Diversity and distribution of begomoviruses infecting tomato in India. Arch. Virol..

[35] Kwok S, Higuchi R (1989). Avoiding false positives with PCR. Nature..

[36] Hall TA (1999). BioEdit: an user-friendly biological sequence alignment editor and analysis program for Windows 95/98/NT. Nucl. Acids. Symp. Ser..

[37] Silva JCF (2017). Geminivirus data warehouse: a database enriched with machine learning approaches. BMC. Bioinformatics..

[38] Altschul SF, Gish W, Miller W, Myers EW, Lipman DJ (1990). Basic local alignment search tool. J. Mol. Biol..

[39] Kuiken C, Yusim K, Boykin L, Richardson R (2005). The Los Alamos HCV Sequence Database. Bioinformatics..

[40] Edgar RC (2004). MUSCLE: multiple sequence alignment with high accuracy and high throughput. Nucleic. Acids. Res..

[41] Tamura K, Stecher G, Peterson D, Filipski A, Kumar S (2013). MEGA 6: Molecular Evolutionary Genetics Analysis version 6.0. Mol. Biol. Evol..

[42] Muhire, B. M., Varsani, A. & Martin, D. P. SDT: A virus classification tool based on pairwise sequence alignment and identity calculation. *PLoS*. *One*. **9**, e108277, 10.1371/journal.pone.0108277 (2014). 10.1371/journal.pone.0108277 PMC4178126 25259891

[43] van der Walt E (2009). Rapid host adaptation by extensive recombination. J. Gen. Virol..

[44] Huson DH, Bryant D (2006). Application of Phylogenetic Networks in Evolutionary Studies. Mol. Biol. Evol..

[45] Woolley SM, Posada D, Crandall KA (2008). A comparison of phylogenetic network methods using computer simulation. PLoS. One..

[46] Martin DP, Murrell B, Golden M, Khoosal A, Muhire B (2015). RDP4: Detection and analysis of recombination patterns in virus genomes. Virus Evol..

[47] Chen T, Tang YF, Zhao R, He ZF, Lu LH (2016). Identification of the cryptic species of Bemisia tabaci transmitting Cotton leaf curl Multan virus. Acta. Phytophyl. Sin..

[48] Siddiqui K, Mansoor S, Briddon RW, Amin I (2016). Diversity of alphasatellites associated with cotton leaf curl disease in Pakistan. Virol. Rep..

[49] Vinoth Kumar R, Singh D, Singh AK, Chakraborty S (2017). Molecular diversity, recombination and population structure of alphasatellites associated with begomovirus disease complexes. Infect. Genet. Evol..

[50] Radhakrishnan G, Malathi VG, Varma A (2004). Biological characterization of an isolate of Cotton leaf curl Rajasthan virus from northern India and identification of sources of resistance. Indian Phytopath..

[51] Kranthi, K. R. Cotton Leaf Curl Virus Time Bomb. *Cotton statistics & News*, http://cicr.org.in/pdf/Kranthi_art/CLCuD_Time_Bomb_ap_2014.pdf (2014).

[52] Akhtar KP (2010). Evaluation of Gossypium species for resistance to cotton leaf curl Burewala virus. Ann. Appl. Biol..

[53] Zhao Y (2016). Bt proteins Cry1Ah and Cry2Ab do not affect cotton aphid *Aphis gossypii* and ladybeetle *Propylea japonica*. Sci. Rep..

[54] Hagenbucher S (2013). Pest trade-offs in technology: reduced damage by caterpillars in Bt cotton benefits aphids. Proc. R. Soc. B..

[55] Gutierrez AP, Ponti L, Herren HR, Baumgärtner J, Kenmore PE (2015). Deconstructing Indian cotton: weather, yields, and suicides. Environ. Sci. Europe..

[56] Venilla S (2008). Pest management for cotton ecosystems or ecosystem management for cotton protection?. Curr. Sci..

[57] FAOStat. Food and Agriculture Organization of the United Nations Statistics Division. http://faostat3.fao.org/home/E (2013).

