# Supplementary material for: Rebound of Cotton leaf curl Multan virus and its exclusive detection in cotton leaf curl disease outbreak, Punjab (India), 2015
Source: Sci Rep. 2017 Dec 12;7:17361. doi: 10.1038/s41598-017-17680-9 (PMC5727119; doi:10.1038/s41598-017-17680-9)

SUPPLEMENTARY INFORMATION

Rebound of *Cotton leaf curl Multan virus* and its exclusive detection in cotton leaf curl disease outbreak, Punjab (India), 2015

Sibnarayan Datta[1,*,+], Raghvendra Budhauliya[1,+], Bidisha Das[1,+], Reji Gopalakrishnan[1,2], Sonika Sharma[1], Soumya Chatterjee[1], Vanlalhmuaka[1], P Srinivas Raju[1] & Vijay Veer[1]

[1]Molecular Virology Laboratory, Biotechnology Division, Defence Research Laboratory (DRL-DRDO), Tezpur, 784 001, INDIA

[2]Present address- Vector Management Division, Defence Research & Development Establishment (DRDE-DRDO), Gwalior, 474 002, INDIA

*sndatta1978@gmail.com

[+]These authors contributed equally to this work.

Table S1. Details of PCR primers used in the present study for amplification of begomovirus genome.

Primer	Orientation	Sequence (5'-3')	Amplicon size (approximate)	Reference
Deng A	Forward	taatattacckgwkgvccsc	550 bp	Chowda Reddy et al., 2005
Deng B	Reverse	tggacyttrcawggbccttca		
PAL1v 1978	Forward	gcatctgcaggcccacatygtcttyccngt	1290 bp	Rojas et al., 1993
PAR1c 496	Reverse	aat act gca ggg ctt yct rta cat rgg		
CLCuV-1F	Forward	cgt aat tat gtc gaa gcg a	950 bp	Designed in this study
CLCuV-4R	Reverse	gac tgg tck ttt ctt aag ggt		
CLCuV-5F	Forward	tat atg gcd tgt act cac gc	1120 bp	
CLCuV-1R	Reverse	atc tag aca gaa tct ttc agg a		

Table S2. Detailed features of the complete alphasatellite sequences generated in the present study.

Alphasatellite species	Number of clones (≥99% PNI)	Representative Isolate No. (GenBank Accession)	Length	Highest scoring BLAST matches; GenBank accession (country, Year of Isolation)	% coverage/ % identity	Rep ORF (frame)/ nts/aa
Tomato leaf curl alphasatellite (ToLCA)	05	C38 MF929026	1377	LN829154 (Pakistan, 2015)<br>LN829155 (Pakistan, 2015)	100/95-97	78-1025 (+3) / 948/315
	02	C39 MF929027	1376			
	01	C40 MF929028	1377			
	03	C46 MF929033	1375			
	03	C47 MF929034	1378			
	02	C48 MF929035	1378			
Okra leaf curl alphasatellite (OLCuA)	03	C31 MF929022	1378	HG934824 (Pakistan, 2013)<br>HE966416 (Pakistan, 2011)<br>HE972285 (Pakistan, 2011)<br>KF471056 (India, 2008)<br>HG934784 (Pakistan, 2013)<br>HG934783 (Pakistan, 2013)<br>LN811059 (Pakistan, 2015)<br>LN810543 (Pakistan, 2015)<br>LN810542 (Pakistan, 2015)<br>HG934784 (Pakistan, 2013)<br>HG934825 (Pakistan, 2013)	100/93-99	80-1027 (+2)/ 948/315
	01	C37 MF929025	1362			
	02	C43 MF929030	1362			
	02	C44 MF929031	1374			
	01	C51 MF929036	1360			
Ageratum yellow vein India alphasatellite (AYVIA)	02	C36 MF929024	1363	HG934821 (Pakistan, 2013)<br>JX570736 (India, 2012)<br>HG515070 (Pakistan, 2012)<br>HG515071 (Pakistan, 2012)	100/95-96	79-1026 (+1)/ 948/315
Gossypium darwinii symptomless alphasatellite (GDarSLA)	02	C35 MF929023	1372	KX656841 (Pakistan, 2015)<br>LN874303 (Pakistan, 2012)<br>KX656842 (Pakistan, 2015)<br>FJ218493 (Pakistan, 2006)<br>KX656840 (Pakistan, 2015)	100/99	70-1017 (+1)/ 948/315
	01	C45 MF929032	1371			

Table S3. Statistical features of recombination events observed in complete alphasatellite sequences analyzed in the present study.

Recombination Event	Recombinant Sequence(s)- this study	Parental Sequence(s)		P-value calculated by different recombination detection methods						
		Minor	Major	RDP	GENECONV	Bootscan	Maxchi	Chimaera	SiSscan	3Seq
R11	MF929027 MF929033 MF929026 MF929028 MF929034 MF929035	KX656849 (GDavSLA) Vehari, Pakistan, 2015	MF929032 (GDarSLA) Punjab, India, 2015	3.29E-04	NS	2.57E-03	1.46E-11	1.95E-05	1.63E-28	5.65E-05
R12	MF929027 MF929033 MF929026 MF929028 MF929034 MF929035	HG934796 (CLCuBuA) Janiwala, Pakistan, 2013	HE966422 (ToLCPKA) Mochiwala, Pakistan, 2011	6.83E-17	6.24E-16	4.08E-12	4.61E-12	3.11E-12	8.67E-14	7.54E-18
R13	MF929022 MF929025 MF929030 MF929036 MF929031	KX656848 (GDavSLA) Vehari, Pakistan, 2015	HE965679 (GDarSLA) Dabar Shah Jivna, Pakistan, 2011	8.44E-06	NS	4.82E-04	5.83E-16	1.14E-07	1.56E-31	8.90E-06
R14#	MF929036 MF929025 MF929022	HG530544 * (CLCuGeA) Jazan, Saudi Arabia, 2013	FN658727 (CLCuBuA) Punjab, India, 2007	NS	NS	NS	2.14E-05	NS	4.32E-10	NS
R15	MF929030	HE965679 (GDarSLA) Dabar Shah Jivna, Pakistan, 2011	KX656848 (GDavSLA) Vehari, Pakistan, 2015	8.44E-06	NS	4.82E-04	5.83E-16	1.14E-07	1.56E-31	8.90E-06
R16	MF929030	FJ218493 (GDarSLA) Pakistan, 2006	LN811059 (OLCuA) Jhang, Pakistan, 2015	1.36E-21	2.98E-25	1.72E-20	1.85E-11	4.72E-12	2.11E-12	9.83E-12
R17	MF929032 MF929031	HG934799 (GDarSLA) Janiwala, Pakistan, 2013	HG934821* (AYVIA) Rajowal, Pakistan, 2013	2.10E-21	5.67E-25	8.68E-15	9.35E-16	1.98E-11	1.55E-20	2.26E-10
R18	MF929024	JX570736* (AYVIA) Lucknow, India, 2012	HG515070 (AYVIA) Kot Jalal, Pakistan, 2012	1.06E-03	NS	NS	8.08E-09	NS	6.00E-06	6.02E-08
R19	MF929032 MF929023	KX656849 (GDavSLA) Vehari, Pakistan, 2015	LN831967 (CLCuMuA) Multan, Pakistan, 2015	2.40E-02	NS	NS	8.65E-11	6.32E-06	1.06E-30	8.03E-03
R20	MF929023	LN870387* (CLCuMuA) Multan, Pakistan, 2015	HG934825 (OLCuA) Janiwala, Pakistan, 2013	9.19E-03	1.37E-02	NS	4.48E-03	NS	NS	1.18E-02

*Parental sequence was unknown; this sequence was used to determine parental sequence
Recombination event detected by two programs

Figure S1. Cotton plants from Punjab displaying typical symptoms of CLCuD.

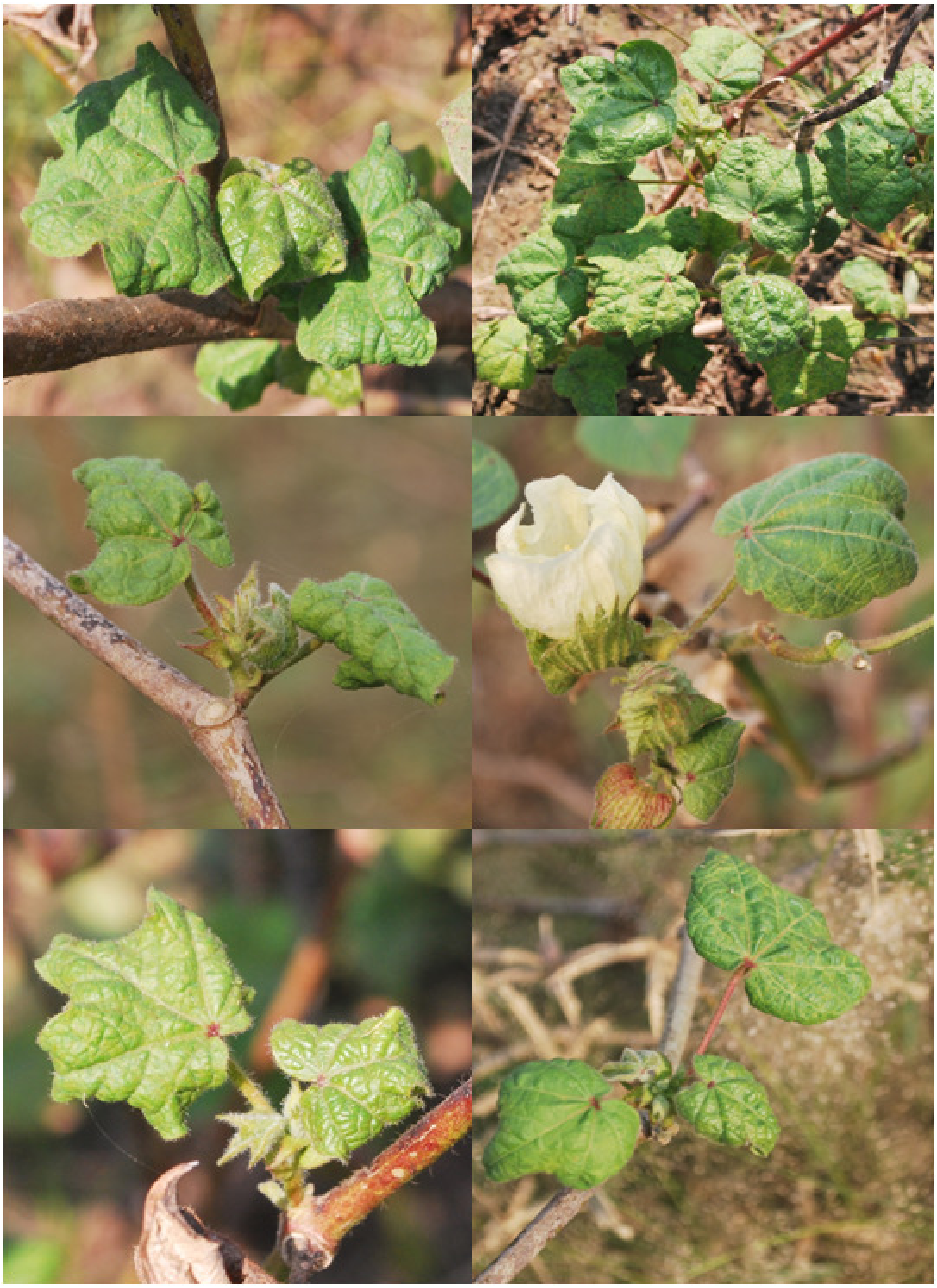

Figure S2. Annealing sites of primers used in this study for complete genome amplification of the begomoviruses.

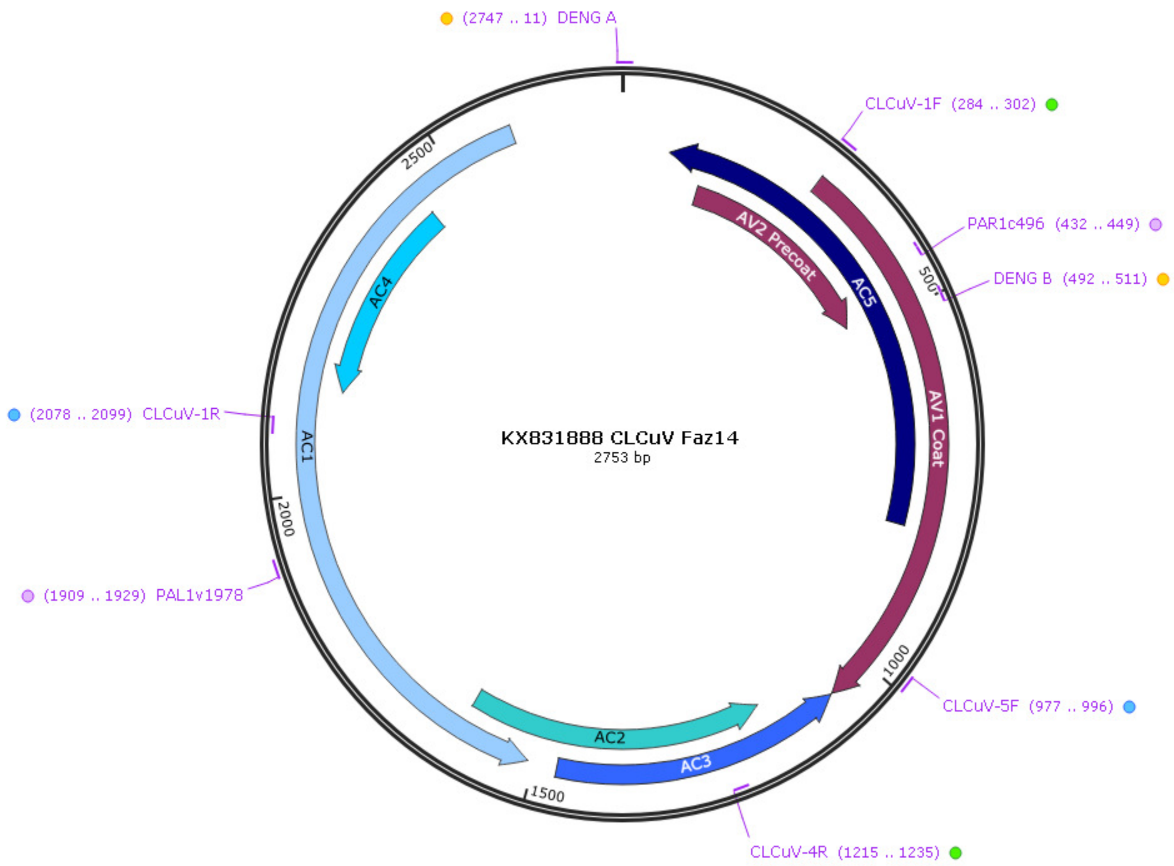

Figure S3. SDT matrix showing the pairwise similarity of the present sequences to CLCuD associated begomovirus (CAB) type species/strains and NCBI RefSeq sequences. Present sequences are indicated within red boxes.

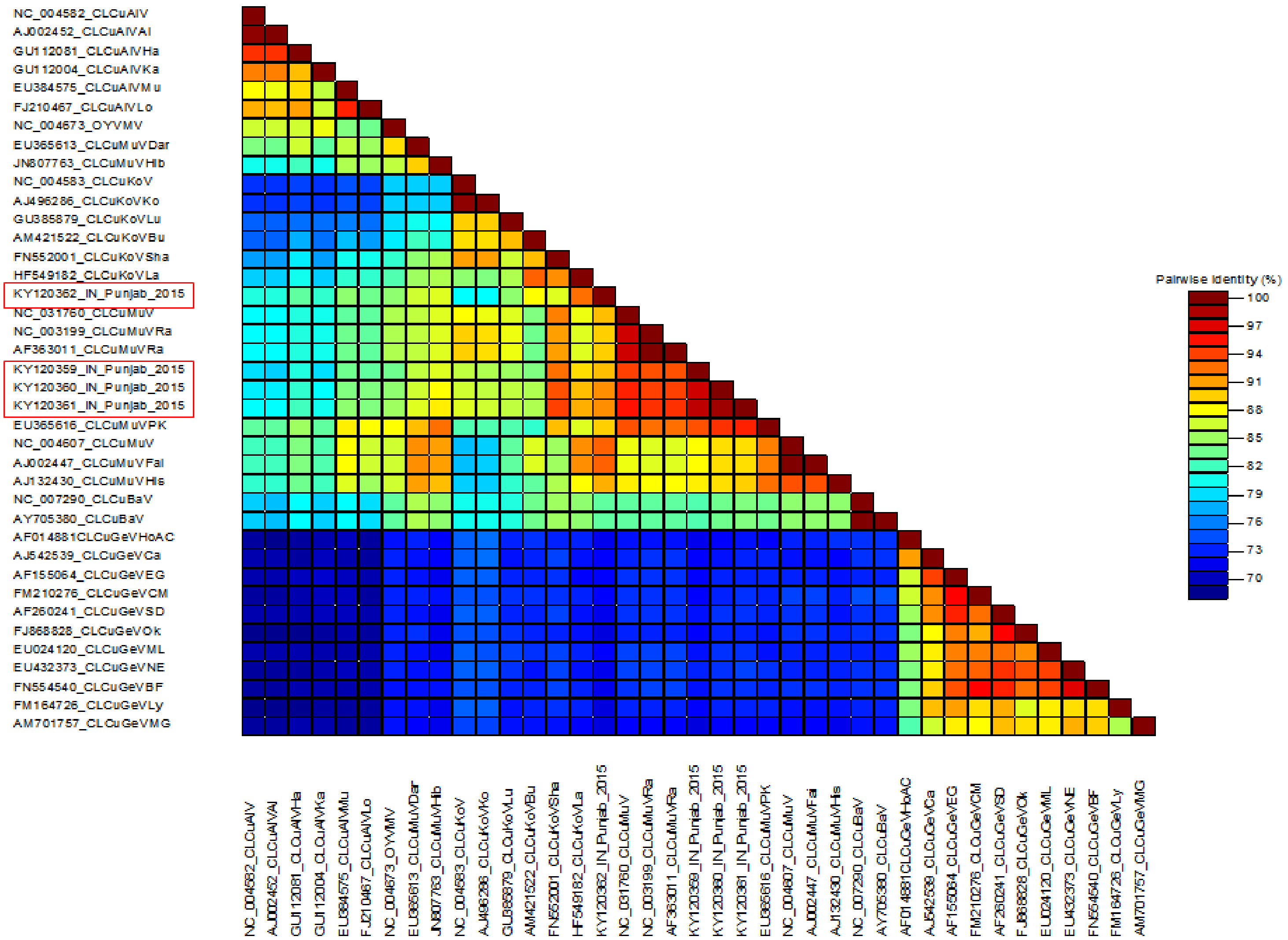

Figure S4. SDT matrix showing pairwise similarity of the present sequences to NCBI RefSeq sequences. Present sequences are indicated within red boxes.

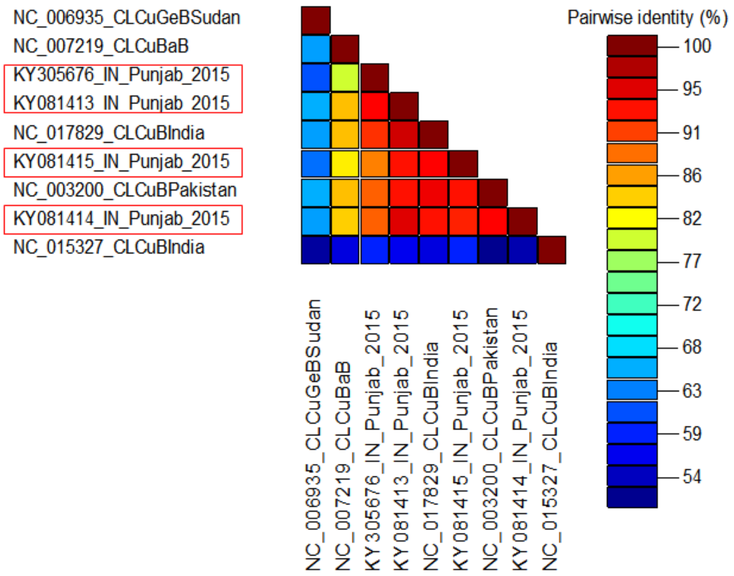

Figure S5. Graphical representation of recombination patterns of the novel CLCuMuV sequences isolated in the present study (KY120359-KY120361) and sequences recently isolated from Vehari, Pakistan (KX656806, KX656809, KX656810). Recombination fragments having same origin are represented by same colours.

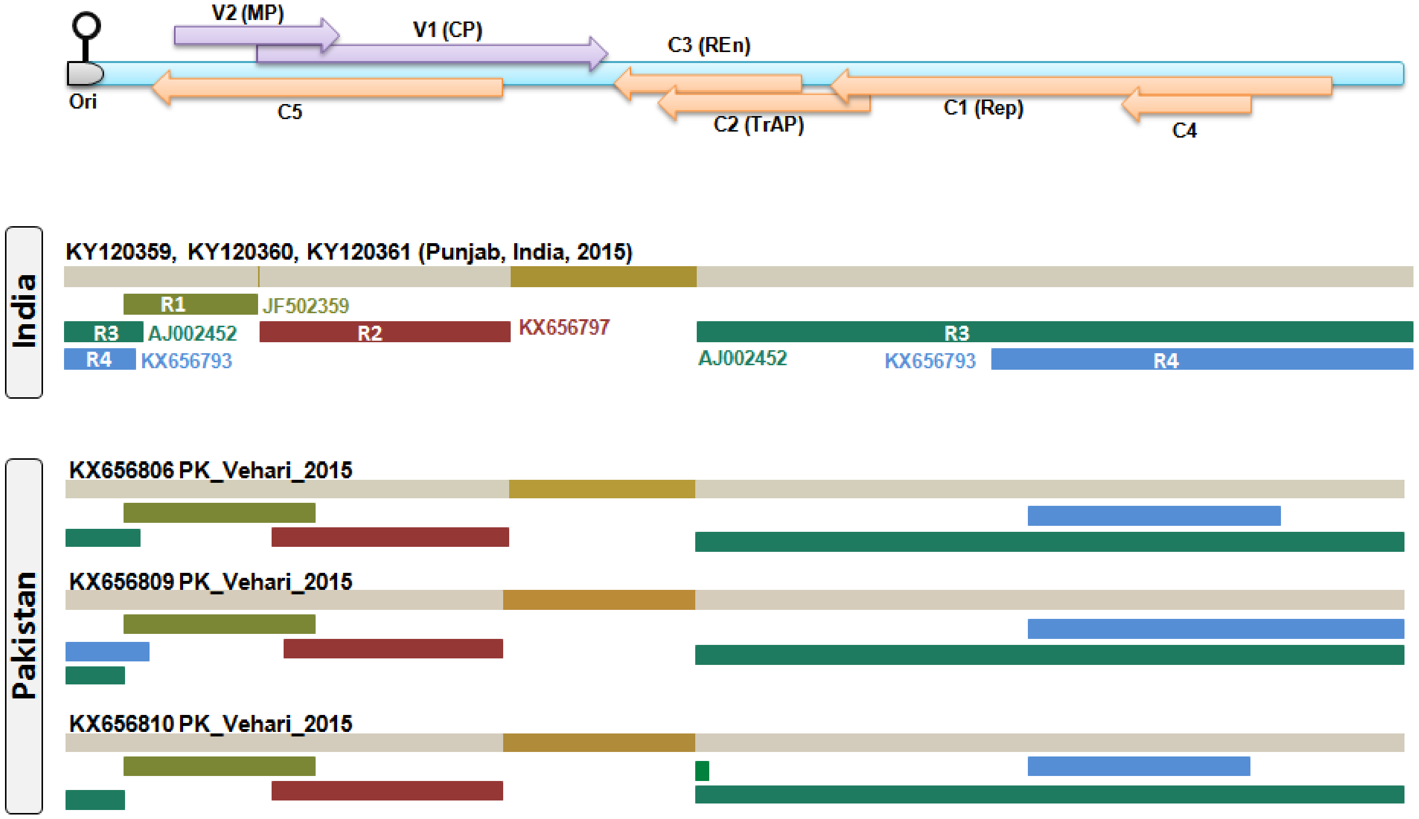

Figure S6. Graphical representation of recombination patterns of the alphasatellite sequences isolated in the present study. Recombination fragments having same origin are represented by same colours. (A) ToLCA, (B) OLCuA, (C) AYVIA, (D) GDarSLA.

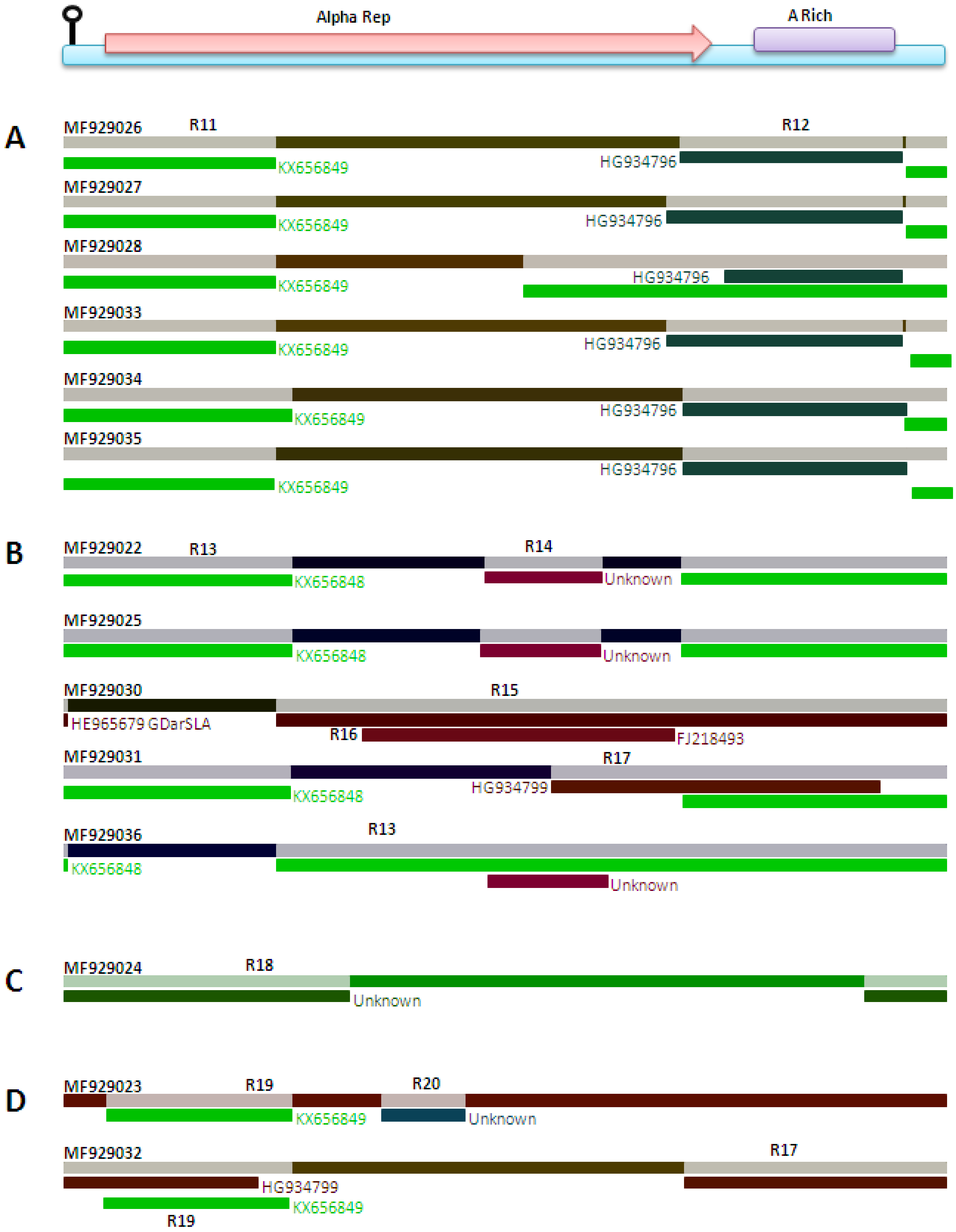

Supplement: Supplementary file 1 — Supplementary Information [file 41598_2017_17680_MOESM1_ESM.pdf]
